# Contrasting Strategies of Alfalfa Stem Elongation in Response to Fall Dormancy in Early Growth Stage: The Tradeoff between Internode Length and Internode Number

**DOI:** 10.1371/journal.pone.0135934

**Published:** 2015-08-17

**Authors:** Zhiying Liu, Xiliang Li, Zongli Wang, Qizhong Sun

**Affiliations:** 1 Institute of Grassland Research, Chinese Academy of Agricultural Sciences, Hohhot, P.R. China; 2 Graduate School of Chinese Academy of Agricultural Sciences, Beijing, P.R. China; 3 Animal husbandry department of Ministry of Agriculture, Beijing, P.R. China; Wageningen University, NETHERLANDS

## Abstract

Fall dormancy (FD) in alfalfa (*Medicago sativa* L.) can be described using 11 FD ratings, is widely used as an important indicator of stress resistance, productive performance and spring growth. However, the contrasting growth strategies in internode length and internode number in alfalfa cultivars with different FD rating are poorly understood. Here, a growth chamber study was conducted to investigate the effect of FD on plant height, aboveground biomass, internode length, and internode number in alfalfa individuals in the early growth stages. In order to simulate the alfalfa growth environment in the early stage, 11 alfalfa cultivars with FD ratings from one to 11 were chosen and seeded at the greenhouse, and then were transplanted into an artificial growth chamber. The experimental design was a randomized complete block in a split-plot arrangement with three replicates. Plant height, above-ground biomass, internode length, and internode number were measured in early growth stage in all individuals. Our findings showed that plant height and the aboveground biomass of alfalfa did not significantly differ among 11 different FD rated cultivars. Also, internode length and internode number positively affected plant height and the aboveground biomass of alfalfa individuals and the average internode length significantly increased with increasing FD rating. However, internode number tended to sharply decline when the FD rating increased. Moreover, there were no correlations, slightly negative correlations, and strongly negative correlations between internode length and internode number in alfalfa individuals among the three scales, including within-FD ratings, within-FD categories and inter-FD ratings, respectively. Therefore, our results highlighted that contrasting growth strategies in stem elongation were adopted by alfalfa with different FD ratings in the early growth stage. Alfalfa cultivars with a high FD rating have longer internodes, whereas more dormant alfalfa cultivars have a larger number of internodes. There were tradeoffs between internode length and internode number in response to FD in alfalfa, which reflected certain scale-dependence.

## Introduction

Plant growth habit is a fundamental characteristic but one that is greatly modified by environmental factors [1]. Habit depends on a combination of different factors, such as internode patterns in the vegetative shoot, leaf traits, leaf to stem ratio, and growing canopy [2–4]. Further, as for internode patterns, plants may differ in internode length and internode number during different growing stages [5].

Internode measurements were used to study the effect of insect injury in alfalfa (*Medicago sativa* L.) [6]. In addition, varietal differences in internode number and internode length were statistically significant in ten common varieties of alfalfa [1]. That technique could be useful in varietal purity testing, genetic studies, and characterization of alfalfa varieties. Alfalfa is the most widely cultivated forage legume in the world due to its high biomass production and high quality. The alfalfa stem is a major organ that affects plant height, above-ground biomass, and forage yield. A stem consists of a series of internodes connected by nodes. The sum of the lengths of elongated internodes accounts for a large fraction of plant height [7]. Internode elongation is influenced by various factors such as growth duration, photoperiod, temperature, and stress inducing environments [8], and involves different signaling pathways. Previous studies on alfalfa stem traits have revealed differences among cultivars in individual internodes, and suggested that the height of plants grown under standardized conditions might be of importance in cultivar purity testing [1].

Fall dormancy (FD), a useful trait that defines alfalfa adaptation to distinct climate conditions [9], is defined as the reduction in shoot growth in the autumn due to decreasing day-length and temperatures [10]. Alfalfa cultivars can be grouped into eleven FD ratings (from one to eleven) based on regrowth height in the autumn, with FD = one representing those going dormant earliest and having the smallest plant height in the fall, and FD = eleven representing those having the tallest plant height in the fall and being the least dormant [10,11]. Cultivars can be further described as fall dormant (FDT, with FD ratings from one to three), semi-fall dormant (SDT, with FD ratings from four to six), non-fall dormant (NDT, with FD ratings from seven to nine), and extremely non-fall dormant (EDT, with FD ratings from ten to eleven [12,13]. Fall dormant cultivars go dormant the earliest, have reduced shoot elongation in the fall and are very winter hardy, whereas EDT cultivars continue to grow, have the tallest plant height in the fall, and generally are not hardy over the winter [14,15]. Therefore, choosing non-dormant cultivars could result in higher forage yield. Historically there has been interest in using less fall-dormant cultivars in regions with mild winters [16,17]. However, research has suggested that no genetic correlation exists between FD and winter hardiness in alfalfa cultivars [18] [19].

Fall dormancy related differences in alfalfa shoot growth have been reported previously [20]. The FD rating, or FD category, has an impact on agronomic performance of alfalfa in several ways, including productivity [9,21], nutritive value [22,23], plant survival [24,25], root characteristics [26], and persistence [11]. As for the internode length and internode number, Sheridan and McKee found differences among alfalfa cultivars for internode number and internode length in the primary stem when the first flowers appeared [1]. The influence of drought on internode number and internode length of alfalfa has been previously reported [27]**.** Previous studies have also aimed to analyze the characteristics of alfalfa stems [28–30]. However, these studies focused on integrative parameters (stem length, cell wall components, etc.), which could not discriminate between the explicit effects of the FD rating defined by the internode length and internode number in the early growing stage. However, little research has been conducted on the influence of FD rating on the internode number and internode length of alfalfa in the early growth stage.

The objective of this study was to explore the growth strategy of eleven alfalfa cultivars with different FD ratings (from one to eleven) in their early growth stage. We addressed the following three questions: (i) How does FD rating affect the plant height and aboveground biomass in alfalfa in the early growth stages? (ii) How does FD rating affect the internode length and internode number? (iii) How do alfalfa cultivars with contrasting FD ratings react to the tradeoff between internode length and internode number? By scaling down our analysis to the internode, this study will provide novel information on the growth strategy of alfalfa.

## Materials and Methods

### Ethics statement

The study was carried out on private land, and the owner of the land gave permission to conduct the study on this site. The field studies did not involve endangered or protected species. And no specific permissions were required for these activities.

### Plant material and cultivation

A single set of check cultivars representing FD ratings one to eleven as described by Teuber et al. [10] are listed in Table 1. These check cultivars have been selected to maintain the intended relationship between the original set of nine check cultivars [10] and to show minimal variation across environments. The nursery stock was established in Wuyuan (40°46′N, 107°35′ E; elevation 1102.7 m) in the midwest of Inner Mongolia, China. In April 2014 in an unheated greenhouse in Wuyuan, alfalfa seeds were sown into standard cultivation pots (top diameter 4 cm, bottom diameter 1.5 cm, height 20.5 cm) filled with a nutrient soil matrix deep enough to allow root development. Each pot contained one or two seeds with 150 pots (50 plants per replication, three replications) per cultivar. Seeds were inoculated with a commercial inoculant of *Sinorhizobium meliloti* Dang at the time of planting. The temperature was 17°C to 26°C and plants were grown following a natural photoperiod. Plants were kept well–watered and weed and insect control was conducted as necessary.

**Table 1:** Cultivars, fall dormancy classes, fall dormancy ratings, fall dormancy types and seed sources of alfalfa evaluated in this study at Wuyuan and Hohhot, Inner Mongolia in 2014. FDC was used by the Certified Alfalfa Seed Council (CASC). FDR represents the actual fall dormancy rating of each check cultivar based on the average University of California regression and the Certified Alfalfa Seed Council Class.

Cultivars	FDC†	FDR‡	FD types
**Maverick**	**1.0**	**0.8**	
**Vernal**	**2.0**	**2.0**	**Dormant**
**5246**	**3.0**	**3.4**	
**Legend**	**4.0**	**3.8**	
**Archer**	**5.0**	**5.3**	**Semi-dormant**
**ABI700**	**6.0**	**6.3**	
**Dona Ana**	**7.0**	**6.7**	
**Pierce**	**8.0**	**7.8**	**Non-dormant**
**CUF101**	**9.0**	**8.9**	
**UC-1887**	**10.0**	**9.9**	
**UC-1465**	**11.0**	**11.2**	**Very non-dormant**

† Number corresponds to fall dormancy class used by the Certified Alfalfa Seed Council (CASC).

‡ Number corresponds to the value calculated using the University of California regression equation.

### Experimental design

After eight weeks of growth the alfalfa plants were transferred to an artificial growth chamber located at the Institute of Grassland Research, Chinese Academy of Agriculture Sciences, Hohhot (40°83′N, 111°73′ E; elevation 1040 m), Inner Mongolia. The remainder of the experiment was conducted in a growth chamber with three replicates. Pots were randomly arranged in the growth chamber in a controlled environment under the following conditions: 16 h photoperiod, 25°C daytime temperature, and 17°C nighttime temperature. The controlled environment was used to ensure uniformity and to minimize uncontrolled sources of variation. The setup of the lamps in the growth chamber was done according to the method given in [31], with some improvements: Ten high-pressure sodium and metal halide lamps with an output of 400 W each (five lamps on either side of the chamber, left and right, respectively) were hung at a distance of two meters above the alfalfa plants. Artificial lighting was provided by a mixture of the ten lamps (Tuopu Instrument co., Ltd, Hangzhou, China) with a photosynthetic photon flux density (PPFD) of 550 umol photons m^−2^ s^−1^ by using a light sensor above the crop to adjust the PPFD. Plants were watered as necessary. Diseases were not observed in the plants and no chemical applications were necessary during the remainder of the study.

### Sampling and measurement

During the growing season, plants were initially harvested with scissors at a 5 cm stubble height at the beginning of flowering according to standard alfalfa management in northern China on 21 June 2014. Before the first cutting, plant height and individual second internode length of the primary stem were measured with a ruler on 20 June 2014. The length of the second internode was measured according to the report by Sheridan and McKee, who found that varieties that did not differ significantly in length at one internode often did so at other internodes except at internode 12 and upward [1]. Individual internode number of the primary stem was counted at the same time. Plant height, aboveground biomass, internode length, and internode number were measured in all plants. After the first cutting, all plants were then individually dried in an oven at 60°C for 48 h and then were weighed to determine the dry mass.

### Statistical analysis

Cultivars were partitioned into terms corresponding to the FD category for each individual. Significant differences in plant height, above-ground biomass, internode length and internode number among alfalfa cultivars with distinct FD ratings were evaluated using one-way analysis of variance (ANOVA). The relationships between plant height and aboveground biomass, and internode length and internode number were tested with Pearson’s correlation method. The relationships between FD ratings or FD categories and plant height, above-ground biomass, internode length, and internode number were tested with Spearman’s correlation method. Significance levels for all analyses were P = 0.01 and P = 0.05. All the statistical analyses were performed using SPSS statistics software (version 16.0; IBM, Armonk, NY). Data was plotted and images were prepared using Sigmaplot software (version12.0; Systat Software, Inc., USA).

## Results

### Plant height and aboveground biomass

**Fig 1:**
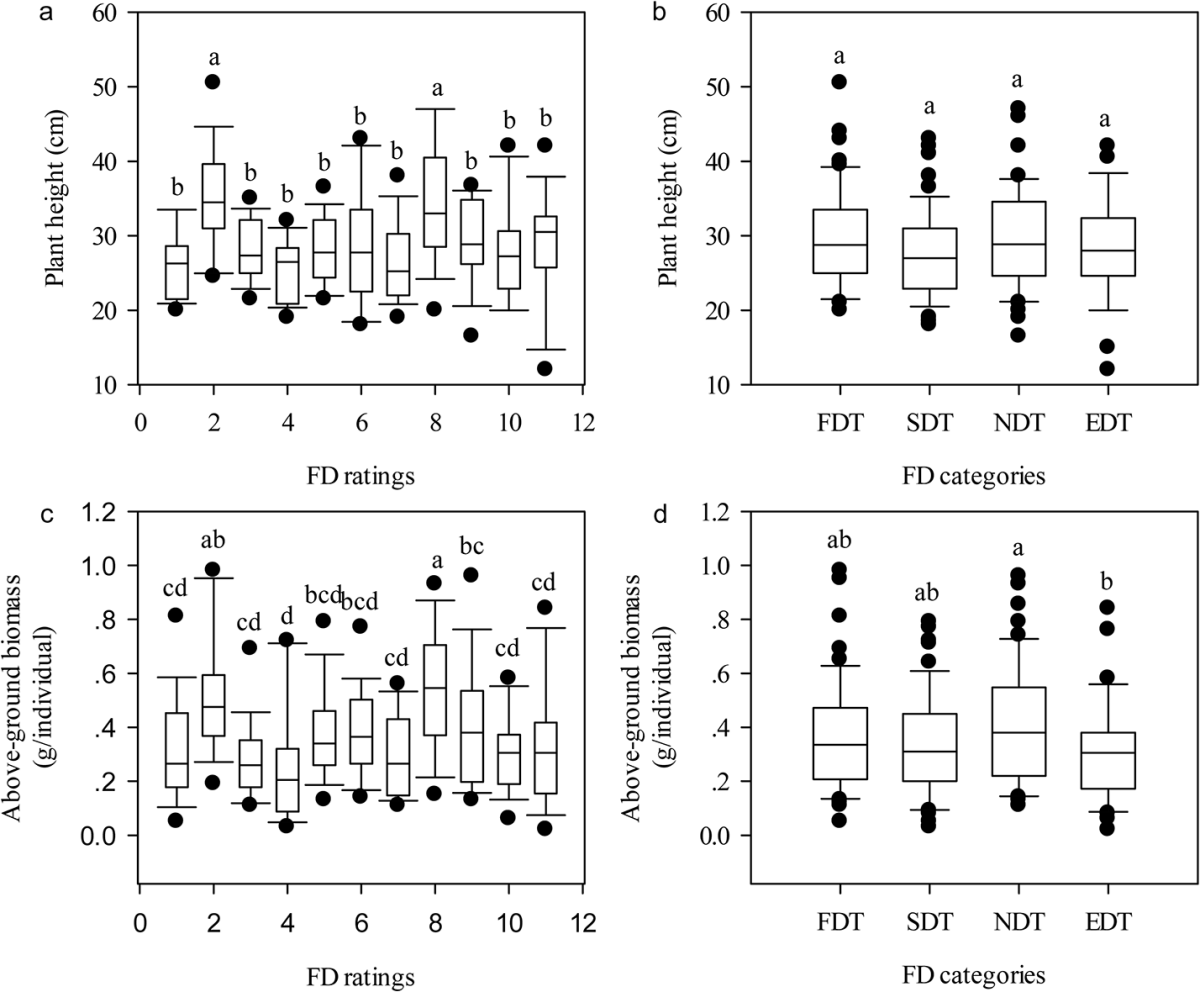
Box plots illustrating the distribution of plant height and above-ground biomass of alfalfa among different FD ratings. (a): Plant height of alfalfa across eleven FD ratings (*r* = -0.02, *P* = 0.83, *n* = 196); (b): Plant height of alfalfa across four FD categories (*r* = -0.03, *P* = 0.67, *n* = 196); (c): Above-ground biomass of alfalfa across eleven FD ratings (*r* = -0.01, *P* = 0.96, *n* = 196); (d): Above-ground biomass of alfalfa across four FD categories (*r* = -0.04, *P* = 0.63, *n* = 196). The relationships of plant height and above-ground biomass with FD ratings were tested by Spearman's rank correlation. Significant differences of FD ratings at 0.05 levels are indicated by different letters. Abbreviations: FD, fall dormancy; FDT, fall dormancy type; SDT, Semi-fall dormancy type; NDT: Non-fall dormancy type; EDT, Extreme non-fall dormancy type.

In the early growth stage, there was no correlation between plant height and FD rating. Though the alfalfa cultivar, Pierce, with FD rating of eight was the tallest, plant height of FD 2 individuals was significantly larger than the other nine cultivars (Fig 1A). Similarly, no significant differences existed among all alfalfa cultivars that belonged to different fall dormancy types (Fig 1B). Although the average aboveground biomass was the largest in the alfalfa cultivar with FD rating of eight, there was little or no differences among other ten alfalfa cultivars with distinct FD ratings (Fig 1C). At the same time, NDT alfalfa cultivars had notably higher aboveground biomass than EDT, FDT, and SDT alfalfa cultivars, whereas no significant differences in the average aboveground biomass existed among FDT and SDT alfalfa cultivars (Fig 1D).

### Internode number in alfalfa cultivars in response to fall dormancy

**Fig 2:**
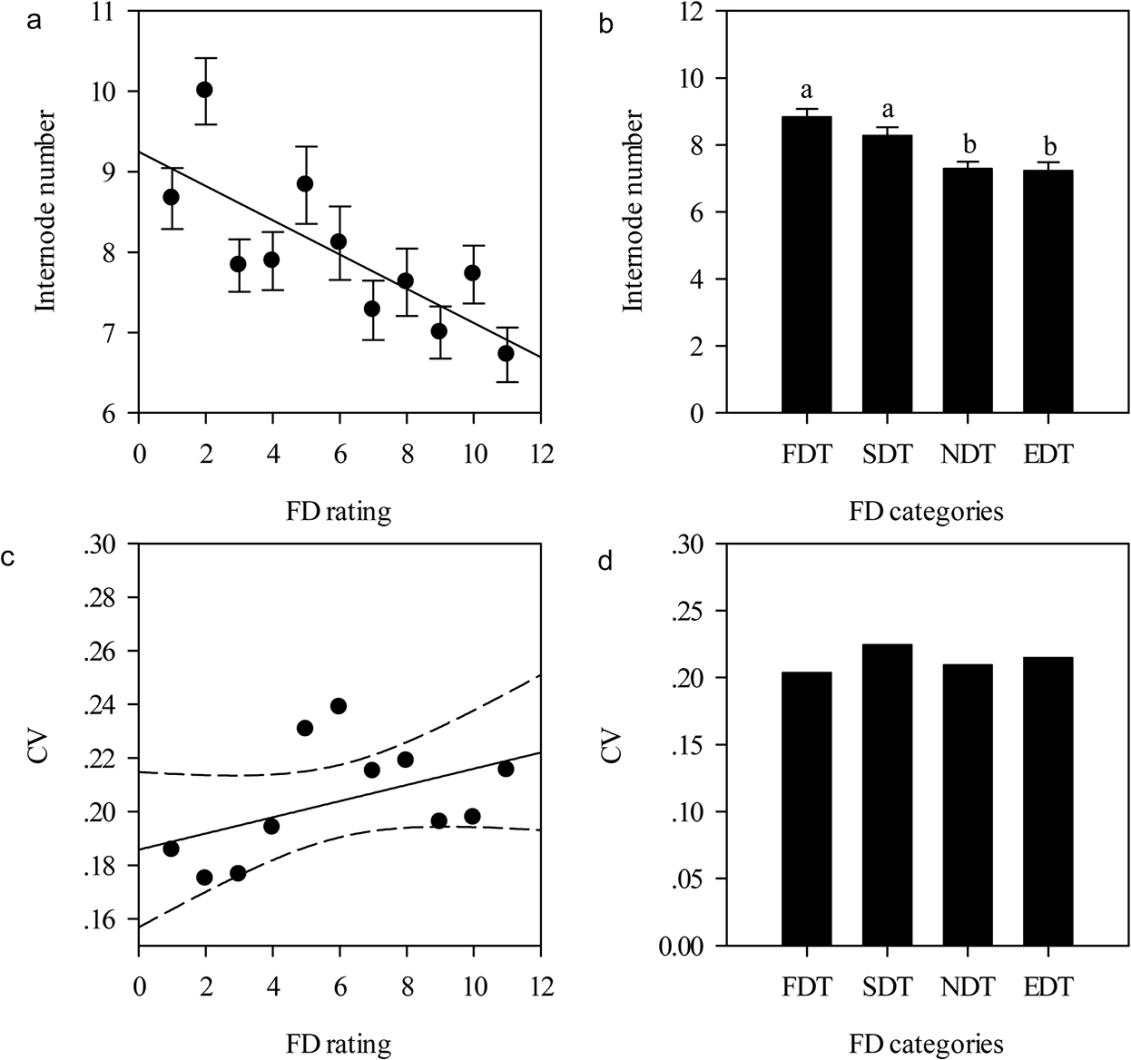
Relationships existing between mean value of the internode number of alfalfa and FD ratings or categories, and between variability of the internode number and FD ratings or categories. (a): Internode number of alfalfa across eleven FD ratings (*r* = -0.27, *P*<0.01, *n* = 196); (b): Internode number of alfalfa across four FD categories; (c): Variation coefficients of internode number of alfalfa across eleven FD ratings (*r* = 0.42, *P*>0.05, *n* = 11); (d): Variation coefficients of internode number of alfalfa across four FD categories (*r* = 0.40, *P* > 0.05, *n* = 4). The relationships between mean value of the internode number of alfalfa and FD ratings or categories, and between variability of the internode number and FD ratings or categories were tested by Spearman's rank correlation. The dashed curve is 95% confidence band. Symbols and abbreviations are the same as in Fig 1. Abbreviations: FD, fall dormancy; FDT, fall dormancy type; SDT, Semi-fall dormancy type; NDT: Non-fall dormancy type; EDT, Extreme non-fall dormancy type.

**Fig 3:**
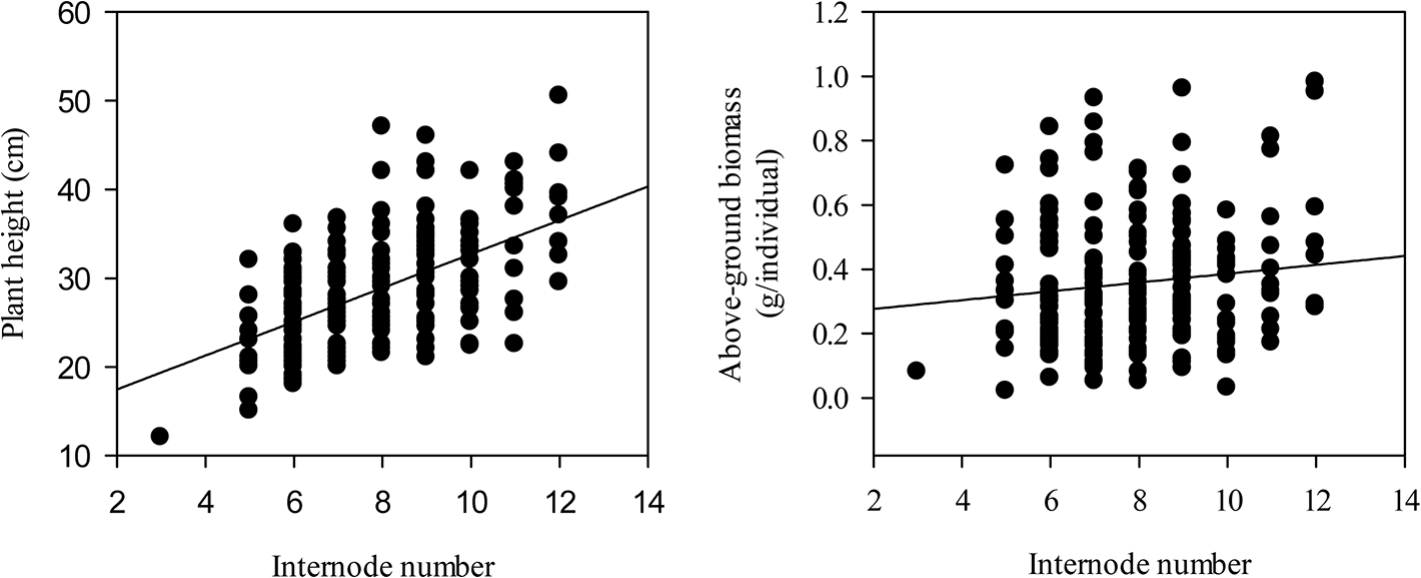
Relationships of plant height, above-ground biomass, and number of internodes of alfalfa. The relationships were tested by Pearson product moment correlations. [Internode number] *vs* [plant height]: *r* = 0.54, *P*<0.001, *n* = 196; [Internode number] *vs* [above-ground biomass]: *r* = 0.12, *P* = 0.08, *n* = 196. Abbreviations: FD, fall dormancy; FDT, fall dormancy type; SDT, Semi-fall dormancy type; NDT: Non-fall dormancy type; EDT, Extreme non-fall dormancy type.

The internode number was affected to varying degrees by FD ratings (Table 2) and FD categories (Table 3). Furthermore, internode number tended to decline sharply when the FD rating increased (Fig 2A), and when alfalfa cultivars became more non-fall dormant (Fig 2B). The degree of variation rose with the increase of the alfalfa FD rating (Fig 2C and 2D). There were positive correlations between the internode number and plant height (*P* < 0.001), yet the average aboveground biomass increased when the internode number increased (Fig 3).

**Table 2:** Statistical characteristics of the internode number of alfalfa across eleven FD ratings.

FD ratings	Minimum value	Maximum value	Mean number	Median number	Variance	Skewness	Kurtosis
**1**	**6.00**	**11.00**	**8.67**	**9.00**	**2.59**	**-0.43**	**-0.71**
**2**	**7.00**	**12.00**	**10.00**	**10.00**	**3.06**	**-0.15**	**-1.43**
**3**	**6.00**	**10.00**	**7.83**	**8.00**	**1.91**	**0.03**	**-1.31**
**4**	**5.00**	**11.00**	**7.89**	**8.00**	**2.34**	**0.21**	**-0.03**
**5**	**5.00**	**12.00**	**8.83**	**9.00**	**4.15**	**-0.03**	**-0.76**
**6**	**5.00**	**11.00**	**8.11**	**8.50**	**3.75**	**-0.07**	**-1.38**
**7**	**5.00**	**11.00**	**7.28**	**7.00**	**2.45**	**0.93**	**0.58**
**8**	**5.00**	**10.00**	**7.63**	**8.00**	**2.78**	**-0.20**	**-1.21**
**9**	**5.00**	**10.00**	**7.00**	**7.00**	**1.88**	**0.61**	**-0.02**
**10**	**5.00**	**11.00**	**7.72**	**7.50**	**2.33**	**-0.14**	**0.31**
**11**	**3.00**	**10.00**	**6.72**	**7.00**	**2.09**	**-0.37**	**2.61**

**Table 3:** Statistical characteristics of the internode number of alfalfa across four FD categories.

FD categories	Minimum value	Maximum value	Mean value	Variance	Skewness	Kurtosis
**Fall dormancy type**	**6.00**	**12.00**	**8.83**	**3.24**	**0.12**	**-0.73**
**Semi-fall dormancy type**	**5.00**	**12.00**	**8.28**	**3.45**	**0.13**	**-0.78**
**Non-fall dormancy type**	**5.00**	**11.00**	**7.29**	**2.33**	**0.45**	**-0.56**
**Extreme non-fall dormancy type**	**3.00**	**11.00**	**7.22**	**2.41**	**-0.15**	**0.90**

### Internode length of alfalfa in response to fall dormancy

**Fig 4:**
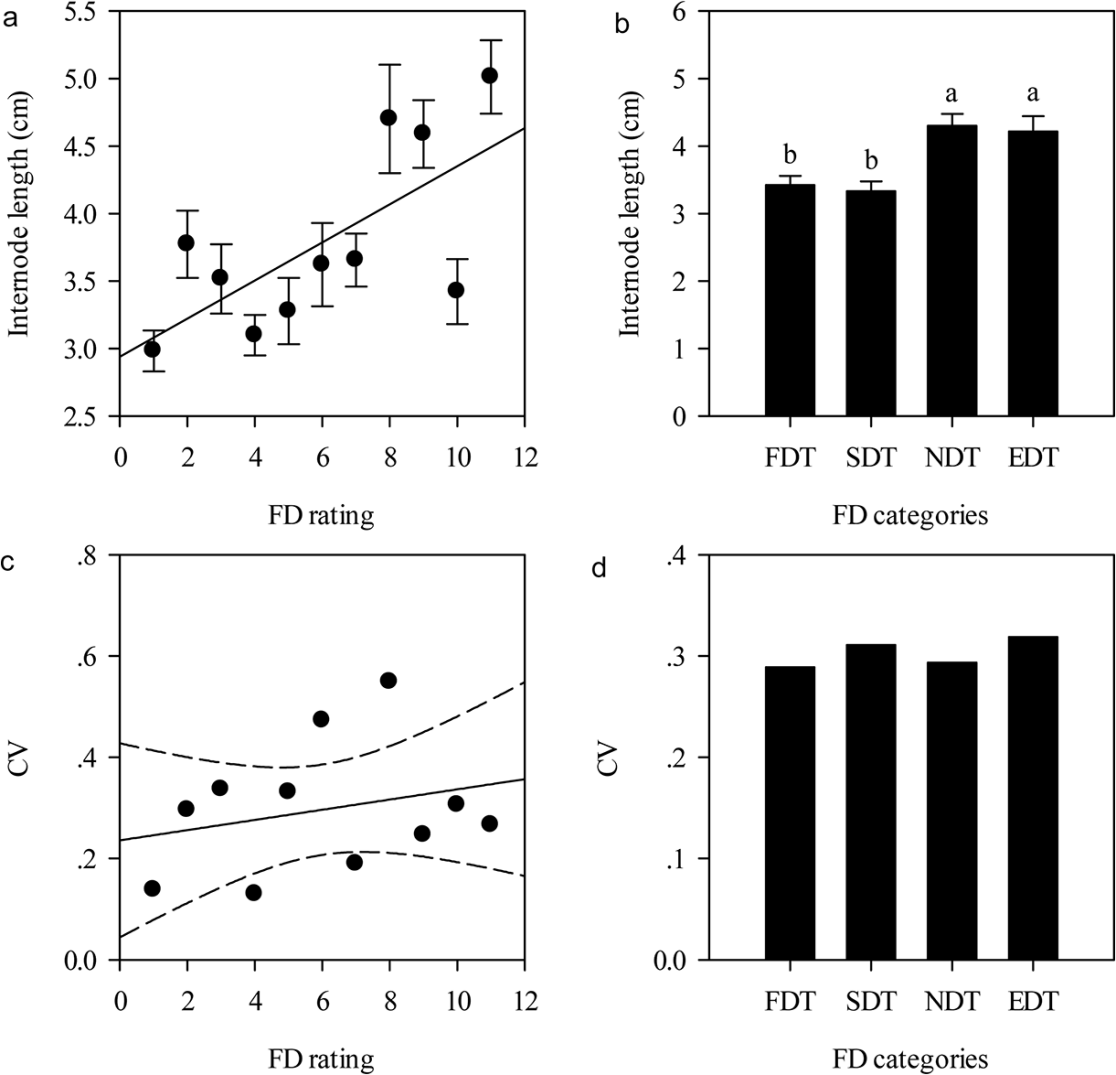
Relationships of the mean value of the internode length of alfalfa and its variability with different fall dormancy ratings. (a): Internode length of alfalfa across eleven FD ratings (*r* = -0.27, *P*<0.01, *n* = 196); (b): Internode length of alfalfa across four FD categories; (c): Variable coefficients of the internode length of alfalfa across four FD categories (*r* = 0.42, *P*>0.05, *n* = 11); (d): Variable coefficients of the internode length of alfalfa across four FD categories. The relationships of mean value of internode length of alfalfa and its variability with FD ratings were tested by Spearman's rank correlation. The dashed curve shows the 95% confidence band. Abbreviations: FD, fall dormancy; FDT, fall dormancy type; SDT, Semi-fall dormancy type; NDT: Non-fall dormancy type; EDT, Extreme non-fall dormancy type.

**Fig 5:**
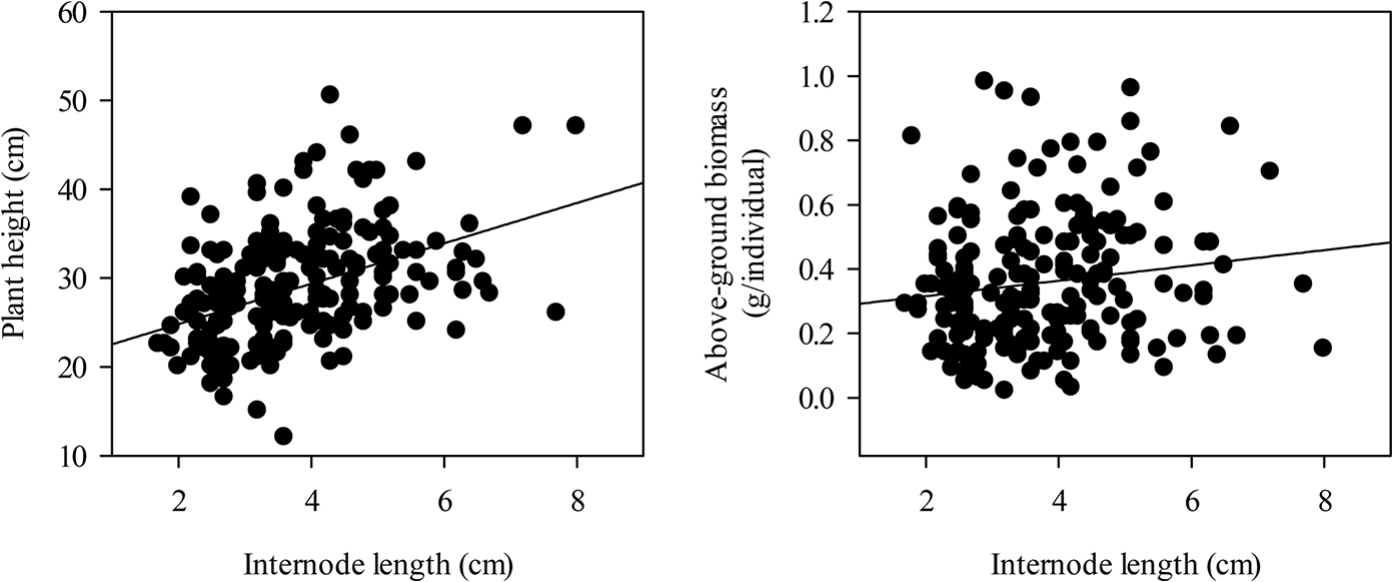
Relationships of plant height and above-ground biomass correlated with the internode length of alfalfa. The relationships were tested using Pearson product moment correlations. [Internode length] *vs* [Plant height]: *r* = 0.43, *P*<0.001, *n* = 196; [Internode length] *vs* [Above-ground biomass]: *r* = 0.15, *P* = 0.04, *n* = 196. Abbreviations: FD, fall dormancy; FDT, fall dormancy type; SDT, Semi-fall dormancy type; NDT: Non-fall dormancy type; EDT, Extreme non-fall dormancy type.

Internode length appeared to be influenced to varying degrees by FD ratings (Table 4) or FD categories (Table 5). Internode length increased as the FD rating increased (*P* < 0.01) (Fig 4A). Additionally, the internode length of non-fall dormant and extremely non-fall dormant alfalfa cultivars were larger than those of fall dormant and semi-fall dormant alfalfa cultivars (Fig 4B). The variable coefficient of the internode length of alfalfa cultivars increased when the FD rating increased (Fig 4C and 4D). There were positive correlations between internode length and plant height (*P* < 0.001), and the average above-ground biomass increased when the internode length increased (Fig 5).

**Table 4:** Statistical test of the internode length of alfalfa across eleven fall dormancy ratings.

FD rating	Minimum value	Maximum value	Mean value	Variance	Skewness	Kurtosis
**1**	**1.80**	**4.10**	**2.98**	**0.41**	**0.09**	**-0.69**
**2**	**2.20**	**5.60**	**3.77**	**1.12**	**0.34**	**-0.80**
**3**	**2.20**	**6.30**	**3.52**	**1.18**	**1.10**	**1.07**
**4**	**2.20**	**4.30**	**3.10**	**0.40**	**0.71**	**-0.55**
**5**	**2.10**	**6.20**	**3.28**	**1.08**	**1.41**	**2.27**
**6**	**1.70**	**6.30**	**3.62**	**1.71**	**0.18**	**-0.65**
**7**	**2.30**	**5.10**	**3.66**	**0.69**	**-0.17**	**-0.82**
**8**	**0.00**	**8.00**	**4.18**	**4.59**	**-0.33**	**0.25**
**9**	**2.70**	**6.70**	**4.59**	**1.13**	**0.23**	**-0.05**
**10**	**2.00**	**5.50**	**3.42**	**1.05**	**0.49**	**-0.79**
**11**	**3.20**	**7.70**	**5.01**	**1.33**	**0.55**	**0.22**

**Table 5:** Statistical test of the internode length of alfalfa across four FD categories.

FD categories	Minimum value	Maximum value	Mean value	Variance	Skewness	Kurtosis
**Fall dormancy type**	**1.80**	**6.30**	**3.42**	**0.98**	**0.87**	**0.47**
**Semi-fall dormancy type**	**1.70**	**6.30**	**3.33**	**1.07**	**0.89**	**0.64**
**Non-fall dormancy type**	**2.30**	**8.00**	**4.30**	**1.59**	**0.74**	**0.61**
**Extreme non-fall dormancy type**	**2.00**	**7.70**	**4.22**	**1.81**	**0.40**	**-0.14**

### Tradeoff between internode length and internode number

**Fig 6:**
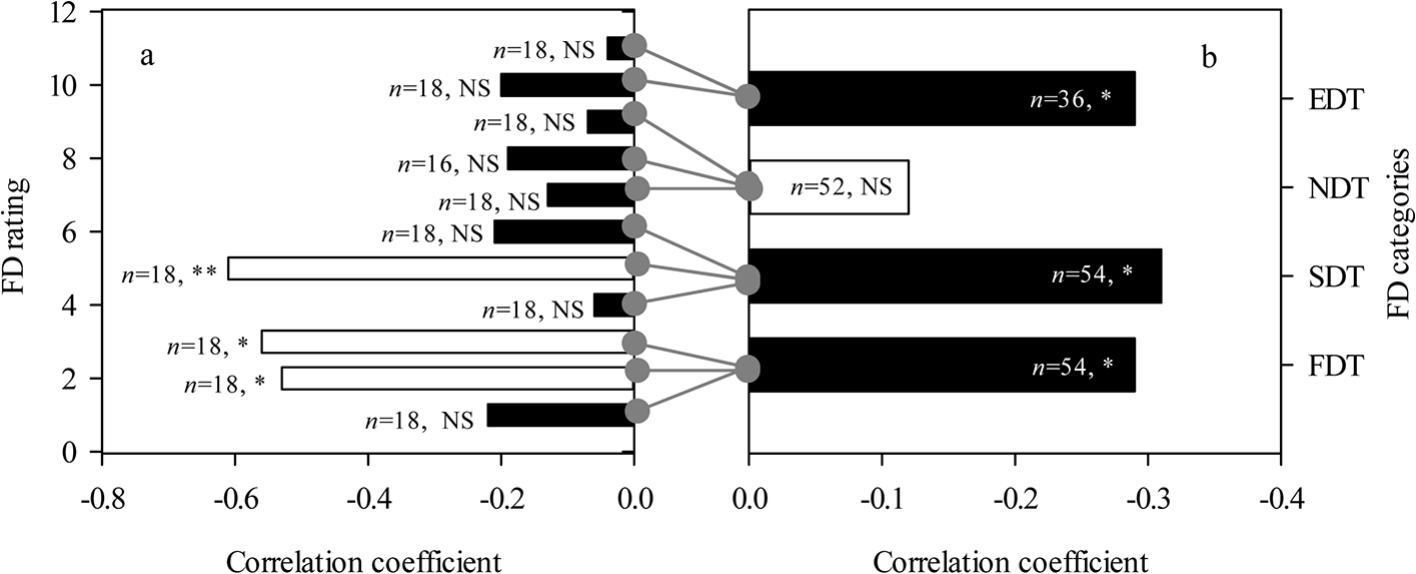
Pearson correlation coefficient between length and number of internode in eleven fall dormancy ratings (a) and four FD categories (b), respectively. Symbols: **, *P*<0.01; *, *P*<0.05; NS, *P*>0.05. Abbreviations: FD, fall dormancy; FDT, fall dormancy type; SDT, Semi-fall dormancy type; NDT: Non-fall dormancy type; EDT, Extreme non-fall dormancy type.

**Fig 7:**
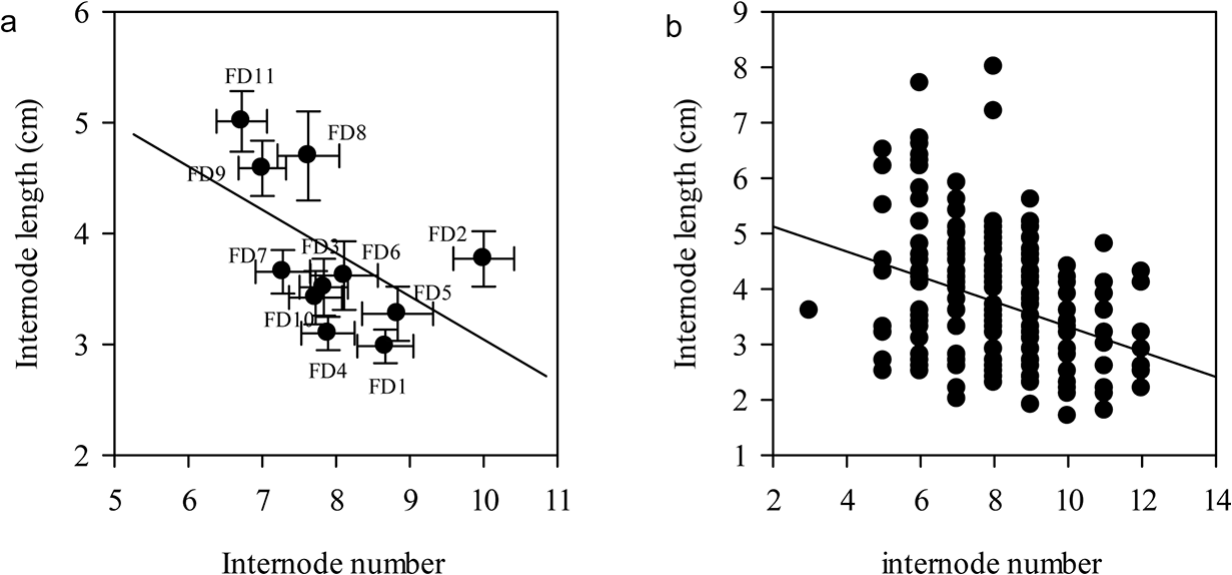
Relationships between internode length and internode number across eleven FD ratings in average (a) and total (b) statistics. The relationships were tested using Pearson product moment correlations. Averaged statistics: *r* = 0.54, *P* = 0.08, *n* = 11; Total statistics: *r = -*0.34, *P*<0.01, *n* = 196. The data were presented by mean ± standard error (SE) in Fig7a. Abbreviations: FD, fall dormancy; FDT, fall dormancy type; SDT, Semi-fall dormancy type; NDT: Non-fall dormancy type; EDT, Extreme non-fall dormancy type.

In the early growth stage a tradeoff between the number of internodes and internode length existed among all alfalfa cultivars. The tradeoff relationship showed negative correlations, which were different under different scales. Within the FD rating scale, the tradeoff characteristics were not obvious, and the majority of FD rated alfalfa cultivars had no correlation between internode number and internode length (*P* > 0.05), except among alfalfa cultivars with FD ratings of two, three, and five (Fig 6A). Within the FD categories scale, FDT, SDT, and EDT alfalfa cultivars had a positive tradeoff relationship between internode number and internode length, but the NDT alfalfa cultivars did not (*P* < 0.05) (Fig 6B). Across eleven FD ratings, internode number and internode length had a strong negative correlation (Fig 7).

## Discussion

We found that plant height and the aboveground biomass of alfalfa cultivars with different FD ratings or FD categories followed similar patterns. The plant height and aboveground biomass of individuals did not differ among alfalfa cultivars with eleven FD ratings or within four FD categories except among those of alfalfa cultivars with an FD rating of eight or FD category of NDT. In NDT alfalfa cultivars, plant height and aboveground biomass were higher than those in FDT, SDT, and EDT alfalfa cultivars. In general, plant height and individual aboveground biomass of alfalfa play an important role in determining forage yield [32]. We speculated that FD rating or FD category of alfalfa might have little effect on forage yield under this condition. This result was similar to the finding reported by Malinowski et al., who demon- strated that FD was unrelated to alfalfa productivity in a rain-fed system in a semiarid/sub-humid environment of the southern Great Plains [22]. Their work also showed that using supplemental irrigation to meet the monthly precipitation during the summer growing season resulted in three to four times greater annual forage yield in comparison with a solely rain-fed system [22]. Consistent with our results, other studies conducted in an environment with temperate winters documented that the choice of FD rating or FD category for alfalfa producers is of secondary importance in comparison to that of harvest regime for forage yield [9,17,32]. In addition, several studies evinced that alfalfa cultivars with a high FD rating were less productive compared to more dormant cultivars [12,13,17], contrary to what was commonly believed [19]. Other possible reasons for our results of no differences between plant height and aboveground biomass of alfalfa cultivars with contrasting FD ratings may also be that severity of drought stress and high temperatures during the growing season might have resulted in the induction of drought-related dormancy in all cultivars regardless of their FD ratings [33]. The specific mechanism of effect of FD rating on alfalfa productivity merits further study.

The ability to manipulate plant growth in the growing season of alfalfa is crucial to managing the balance between harvest regimes and determining the forage yield [9]. The patterns of internode length and internode number were distinct among alfalfa cultivars with different FD ratings or FD categories. Alfalfa cultivars with high FD ratings (weak fall dormancy) tended to have a longer internode length, whereas internode number increased as the FD ratings of alfalfa cultivars decreased. Compared to previous studies the present results further confirm that internode length and internode number are strongly related to the FD rating or the FD category, and this relationship is likely to be dependent on light capture, as reported by Barillot et al., whose simulations based on wheat-pea mixtures showed that internode length was found to be highly sensitive to light partitioning [34]. Cici et al. also reported that changes in the internode length of the chickpea (*Cicer arietinum* L.) competed with phyllochron variations [35]. Interestingly, Lemerle et al. suggested that wheat (*Triticum aestivum* L.) plants with longer internodes had enhanced competitive ability for light capture versus weeds [36]. Therefore, we speculate that alfalfa cultivars with a high FD rating (weak fall dormancy) have longer but fewer internodes than alfalfa cultivars with lower FD ratings, thus alfalfa with a high FD rating can capture more light.

In the present study, internode length increased significantly as the FD rating increased or the alfalfa cultivar became more non-fall dormant, whereas fall dormant alfalfa cultivars had the greatest internode numbers. However, Jung and Lamb observed a similar number of elongating internodes at the time of harvest between the field and growth chamber experiments [37]. One possible reason is that alfalfa cultivars with high FD ratings had higher accumulation of endogenous hormones, such as Gibberellins (GAs) in the stem. The internode elongation seen in plants appears to be mediated by changes in vegetative endogenous hormone levels [38–40]. GAs are potent promoters of stem elongation and have been shown to stimulate expression of beta-expansins. Beta-expansins are related to internode elongation in barley (*Hordeum vulgare*) [41]. GAs also play an important role for internode elongation in Avena [42], Pisum [43], and *Zea mays* L. [44]. Two growth-active GAs (GA1 and GA4) have been shown to mediate a variety of light responses including stem growth (see review [45]). Previous studies also found that GA3 content increased as FD ratings of alfalfa cultivar increased [46]. Furthermore, `in rice stem the elongated uppermost internode (EUI) functions as a GA-deactivating enzyme and the expression of Eui as a GA catabolism gene appears tightly regulated during plant development [7,47]. It is also likely that changes in the leaf content of GAs were best correlated with the enhanced growth of the subtending internode [48], leading to internode length variation among alfalfa cultivars with different FD ratings. Our results may also be caused by strigolactone (SLs), which act independently from GAs to stimulate internode elongation [49].

There is also evidence that internode length is primarily controlled by the shoot genotype [50]. Another study suggested that PIF4 (Phytochrome Interacting Factor 4) causally affects early internode lengths on the primary inflorescence in *Arabidopsis thaliana* [51]. In addition, with the development of molecular biology techniques, major QTLs regulating early internode length and number in alfalfa may be studied in the future. Furthermore, our research implicates that specific plant organs in the fall dormancy response of alfalfa may provide a new insight for studying the metabolic and genetic basis of fall dormancy.

Our results also indicated that alfalfa cultivars with a high FD rating have a longer internode, whereas more dormant alfalfa cultivars have a larger number of internodes in the early growth stages. Further, we concluded that there were tradeoffs between internode length and internode number of alfalfa in response to FD. Interestingly, there were no correlations, slightly negative correlations, and strongly negative correlations between internode length and internode number in alfalfa individuals among the three scales, including within-FD ratings, within-FD categories and inter-FD ratings, respectively. Therefore certain scale-dependence existed in the tradeoff between internode length and internode number. Our results demonstrated that contrasting growth strategies in stem elongation were adopted by alfalfa with different FD ratings in the early growth stages.

## Conclusion

From the results we concluded that alfalfa had contrasting growth strategies of stem elongation with different FD ratings in early growth stage. Alfalfa cultivars with a high FD rating have longer internodes, whereas more dormant alfalfa cultivars have a larger number of internodes. There were tradeoffs between internode length and internode number in alfalfa in response to FD, and this had a certain scale-dependence.
